# Beyond Exposure Levels: Oxidative Stress as a Unifying Axis Linking Particulate Matter, Light, and Protective Interventions

**DOI:** 10.3390/antiox15020236

**Published:** 2026-02-11

**Authors:** Yasuhiro Yoshida

**Affiliations:** Department of Immunology and Parasitology, School of Medicine, University of Occupational and Environmental Health, Japan, 1-1 Iseigaoka, Yahatanishi-ku, Kitakyushu 807-8555, Japan; freude@med.uoeh-u.ac.jp

Air pollution remains one of the most pervasive environmental health threats worldwide, contributing substantially to the global burden of respiratory, cardiovascular, and metabolic diseases. Among various pollutants, fine particulate matter (PM_2.5_) has attracted particular attention because of its ability to penetrate deep into the respiratory tract and induce oxidative stress and inflammation. Importantly, accumulating evidence indicates that the health effects of PM_2.5_ cannot be explained solely by particle mass or exposure concentration. Instead, the quality of exposure—including chemical composition, oxidative potential, and host susceptibility—critically determines biological outcomes [1].

This Special Issue “Oxidative Stress Induced by Air Pollution, 2nd Edition” brings together six original research articles and two comprehensive reviews, all converging on oxidative stress as a central biological axis linking environmental exposure to inflammation, tissue injury, and disease modification across multiple organs.

A central contribution of this Issue is the demonstration that PM_2.5_ is not a uniform toxicant, but rather a complex and dynamic mixture whose biological effects are dictated by its chemical composition. In this Issue, Wang et al. show that seasonal variation in PM_2.5_ composition profoundly shapes pulmonary immune responses using a two-year continuous sampling approach combined with in vivo exposure models [Contribution 1]. Their study demonstrates that PAH-rich PM_2.5_ preferentially induces robust ROS generation and neutrophilic inflammation, whereas mineral-enriched winter PM_2.5_ strongly correlates with IL-1α production, an epithelial- and macrophage-derived alarmin that initiates innate immune activation.

These findings extend prior observations that particle-bound organic compounds drive oxidative stress and mitochondrial dysfunction [2], while mineral and crystalline components can induce cell death-associated alarmin and inflammasome activation [3]. Importantly, the partial attenuation of inflammatory responses in TLR4-deficient mice reported by Wang et al. highlights the limitations of single-receptor models and supports a broader framework in which oxidative stress, cellular injury, and danger signal release jointly orchestrate PM_2.5_-induced inflammation [Contribution 1].

While the lung is the primary portal of entry for inhaled pollutants, several studies in this Issue emphasize that oxidative stress propagates beyond the respiratory system. Miranda-Martínez et al. demonstrate that chronic low-dose ozone exposure disrupts intestinal barrier integrity and induces sustained inflammatory responses along the gastrointestinal tract, supporting the emerging concept of a lung–gut axis in environmental disease [Contribution 2].

Complementing these experimental findings, Santibáñez et al. report that the oxidative potential of personally collected particulate matter is strongly associated with systemic inflammatory markers, including IL-6 and the IL-6/IL-10 ratio, with particularly pronounced effects observed in individuals with asthma [Contribution 3]. These data are consistent with previous epidemiological and mechanistic studies linking particulate oxidative potential to systemic inflammation and cardiovascular risk [4].

The two review articles in this Issue further extend the systemic perspective. Grifoni et al. summarize evidence connecting PM-induced oxidative stress and inflammation to coronary artery disease [Contribution 4], framing particulate matter, oxidative stress, and cardiovascular pathology as a “dark triad.” Lee et al. review emerging data linking PM_2.5_ exposure to prostate cancer risk, highlighting oxidative stress and endocrine-disrupting chemicals as potential mechanistic mediators. Together, these contributions underscore that air pollution should be regarded as a systemic risk factor rather than a lung-restricted hazard [Contribution 5].

Several studies in this Issue explore strategies to mitigate pollution-induced oxidative stress and inflammation [Contribution 6]. Lee et al. demonstrate that thyme leaf extract dose-dependently attenuates PM_2.5_-induced pulmonary injury by reducing ROS generation, inflammatory cytokine production, and mucus hypersecretion while preserving antioxidant defenses. Similarly, Kim et al. report that a combined extract of Dioscorea bulbifera and Zingiber officinale mitigates PM_2.5_-induced respiratory dysfunction through modulation of NF-κB, MAPK, and TGF-β/Smad signaling pathways, ultimately suppressing inflammation and fibrosis [Contribution 7].

Beyond pharmacological and nutritional approaches, Park et al. introduce photobiomodulation as a non-invasive physical intervention capable of attenuating PM_2.5_-exacerbated allergic asthma [Contribution 8]. Their findings demonstrate that light-based therapy suppresses oxidative stress, immune cell infiltration, airway remodeling, and multiple forms of regulated cell death, including ferroptosis. These results align with emerging concepts that host-directed interventions may complement exposure reduction strategies [5].

Collectively, the studies in this Issue converge on a critical insight: oxidative stress represents a shared biological currency through which diverse environmental exposures interact with host immunity and tissue homeostasis. However, the downstream consequences of oxidative stress are highly context dependent, shaped by particle composition, exposure duration, genetic background, and the availability of protective modifiers.

Future research should prioritize integrative approaches that combine detailed exposure characterization with molecular, immunological, and clinical profiling. Such strategies will be essential for advancing precision environmental health, identifying susceptible populations, and developing tailored interventions that enhance resilience against pollution-related diseases.
